# Cardiac dysfunction in type II diabetes: a bittersweet, weighty problem, or both?

**DOI:** 10.1007/s00592-016-0911-8

**Published:** 2016-10-01

**Authors:** Melissa Leung, Vincent W. Wong, Ertugrul Durmush, Victoria Phan, Mikey Xie, Dominic Y. Leung

**Affiliations:** 1 Department of Cardiology, Liverpool Hospital, South Western Sydney Clinical School, University of New South Wales, Locked Bag 7103, Liverpool BC, NSW 1871 Australia; 2 Ingham Institute for Applied Medical Research, Sydney, NSW Australia; 3 Leiden University Medical Centre, Leiden, The Netherlands; 4 Liverpool Diabetes Collaborative Research Unit, South Western Sydney Clinical School, University of New South Wales, Locked Bag 7103, Liverpool BC, NSW 1871 Australia; 5 The Life Weight Loss Centre, Liverpool, NSW Australia

**Keywords:** Diabetic cardiomyopathy, Glycaemic control, Left ventricular function, Echocardiography, Strain, Weight loss, Bariatric surgery

## Abstract

**Aims:**

Weight loss in obese patients leads to improved left ventricular (LV) function. It is unclear whether improving glycaemic control has additional benefits to weight loss alone in patients with type 2 diabetes, or if benefits of weight loss are mediated through improving glycaemic control. This case–control study examined the incremental impact of these approaches on LV function.

**Methods:**

Three groups of age, gender, and baseline HbA1c-matched patients with type 2 diabetes and suboptimal glycaemic control were followed-up for 12 months. Group 1 patients did not improve HbA1c ≥ 1 % (10.9 mmol/mol) or lose weight. Group 2 improved HbA1c ≥ 1 % but did not lose weight. Group 3 improved HbA1c ≥ 1 % (10.9 mmol/mol) and lost weight. All patients underwent transthoracic echocardiogram at baseline and at follow-up.

**Results:**

At baseline, three groups were comparable in all clinical and metabolic parameters except Group 3 had highest body mass index. The three groups had similar echocardiographic parameters except Group 3 had the worst LV systolic function [global longitudinal strain (GLS)]. At follow-up, LV ejection fraction and diastolic function improved with a reduction in filling pressures in Group 2 and more so in Group 3. LV filling pressures in Group 1 increased. There was a significant improvement in GLS in Group 2 and more so in Group 3. Despite GLS being the worst in Group 3 at baseline, this was comparable between Groups 2 and 3 at follow-up.

**Conclusions:**

In overweight patients with type 2 diabetes, weight loss and improved glycaemic control had additive beneficial effects on improving LV systolic and diastolic function.

## Introduction

Patients with type 2 diabetes are often overweight and have multiple vascular risk factors. This may manifest as left ventricular (LV) systolic and/or diastolic dysfunction, or blunted heart rate variability, not attributable to hypertension or myocardial ischaemia [1–5]. The underlying pathophysiologic mechanisms of LV dysfunction in these patients are multifactorial, but hyperglycaemia is considered a main determinant [6, 7]. However, the relationship between glycaemic control and cardiac function has been conflicting with some studies showing poor glycaemic control was associated with abnormal LV relaxation, elevated LV filling pressures, and lower systolic strain [2, 8, 9], while other studies demonstrated no such significant association [10–14]. Furthermore, studies examining the relationship between glycaemic lowering and LV function also gave inconsistent results [15–18].

Echocardiographic strain imaging and tissue Doppler velocities are proven techniques in the assessment of LV systolic and diastolic function that have incremental prognostic value to traditional echocardiographic parameters like LV ejection fraction [19–22]. Echocardiography-based calibrated integrated backscatter (cIB), shown to be related to histologically quantified collagen accumulation, may be used as a measure of myocardial interstitial fibrosis [23, 24].

Obesity alone, in the absence of diabetes, has also been linked to LV dysfunction, and weight loss in obese patients has been shown to result in improved LV function [25]. To date, studies examining the relationship between glycaemic lowering and LV function in patients with type 2 diabetes have not clarified the differential impact of weight loss and improved glycaemic control in overweight patients with type 2 diabetes. It is unclear whether weight loss has incremental benefits to improved glycaemic control in these patients or indeed whether the benefits of weight loss in obese patients with type 2 diabetes are mediated through improved glycaemic control only. This case control study was conducted to examine the differential and incremental benefits of weight loss and improved glycaemic control on LV function in obese patients with type 2 diabetes.

## Subjects, materials, and methods

### Patients

The study patients were recruited from a cardio-diabetology clinic where patients with poorly controlled type 2 diabetes were referred and jointly managed by an endocrinologist and a cardiologist. Eligible patients included adults with type 2 diabetes with suboptimal glycaemic control (glycated haemoglobin [HbA1c] HbA1c ≥ 7.0 %, 53 mmol/mol). Patients with type 1 diabetes, known congenital, valvular, or coronary artery disease (CAD), severe hypertension (>200/120 mmHg at rest), left bundle branch block, rhythm other than sinus, previous or current treatment with thiazolidinediones, and previous history of hypoglycaemia unawareness, were excluded. All patients had significant CAD excluded by exercise echocardiography when they first attended the clinic. Their glycaemic control, blood pressure, and lipid profile were optimized, and they were reviewed 3 monthly for 12 months after which time the patients were discharged back to their primary care physicians. Patients were advised to follow a diet of low glycaemic index and to reduce excessive carbohydrate and fat intake. Overweight or obese patients were recommended a healthy balanced diet but aimed at reduced energy intake and referred for individual dietitian consultation when necessary. Ten patients elected to undergo bariatric surgery of their own volition and were deemed suitable by a bariatric surgeon for laparoscopic sleeve gastrectomy. These patients undertook a pre-operative very low calorie diet in combination with a 3-week exercise programme supervised by an exercise physiologist, a dietician, and a psychologist; following which surgery was performed. All subjects had normal resting electrocardiogram and provided written informed consent. The study was approved by the Hospital Human Ethics Committee.

A group of 20 patients (“Group 1”) who failed to improve (or had worsened) their glycaemic control and did not lose any weight after 12 months was identified. Failure to improve glycaemic control was defined as an improvement of HbA1c of ≤ 1 % (10.9 mmol/mol) or any increase in HbA1c after 12 months. A second group (“Group 2”) of 20 age-, gender-, and baseline HbA1c-matched patients who improved their glycaemic control but did not lose any weight in the 12-month period was selected. The third group (“Group 3”) of ten similarly matched patients at study entry comprised those patients who underwent sleeve gastrectomy, improved their glycaemic control [defined as a decrease of HbA1c > 1 % (10.9 mmol/mol) at 12 months] and who had lost weight over the 12-month period. Therefore, the total study population comprised of 50 patients.

### Baseline clinical and metabolic data

Clinical data collected at baseline included age, height, weight, waist and hip circumference, cardiac risk factors, duration of diabetes, medications, and presence of macrovascular and microvascular complications. All subjects ranked their degree of breathlessness from 1 to 5 using the Medical Research Council (MRC) dyspnea scale [26]. Those ranked scale 1 had minimal dyspnea except on strenuous exercise, while those in scale 5 were too breathlessness to perform simple tasks such as undressing.

Patients’ haemoglobin, HbA1c, serum creatinine, estimated glomerular filtration rate (eGFR) using the Chronic Kidney Disease Epidemiology Collaboration (CKD-EPI) formula [27], fasting total cholesterol, low-density lipoprotein cholesterol (LDL-C), high-density lipoprotein cholesterol (HDL-C), and triglycerides, C-reactive protein (CRP), and urinary spot albumin-to-creatinine ratio were measured at baseline and 12 months.

### Follow-up

All patients were reviewed 3 monthly at the clinic and had their medical treatment for diabetes, blood pressure, and cholesterol levels optimized aiming to achieve guideline-recommended targets. Treatment was maintained and monitored for 12 months.

### Echocardiography protocols

All patients underwent rest echocardiography followed by symptom-limited exercise echocardiography at baseline. A repeat echocardiogram was performed at 12 months.

#### Two-dimensional and Doppler echocardiography

All transthoracic echocardiograms were performed with Vivid E9, GE Medical Systems. All standard echo and Doppler parameters of LV systolic and diastolic function including pulse wave tissue Doppler were measured.

#### Left ventricular strain imaging

Global mean peak longitudinal strain (GLS) and strain rate of the left ventricle was obtained with two-dimensional speckle tracking analyses in apical 4-, 2-chamber and long-axis views using highest possible frame rates. Analyses were performed by experienced observers blinded to the clinical history and metabolic profiles. The GLS and strain rate were calculated from the three global longitudinal strain curves of the three apical views. All Doppler and 2D speckle tracking echocardiographic measurements were taken as averages of three representative cycles.

#### Left atrial volume and function assessment

Left atrial (LA) volumes were measured from the apical views according to the biplane Simpson’s method. The following indices of LA function were measured: LA reservoir volume was calculated as the difference between the maximum and minimum LA volumes. Passive LA emptying volume was calculated as the difference between maximal and pre-contraction LA volumes. Active LA emptying volume was calculated as the difference between pre-contraction and minimum LA volumes. All LA volumes were indexed to body surface area.

#### Calibrated integrated backscatter

The cIB curves were extracted in the parasternal long-axis view, using standard software (Echopac, GE Vingmed). Measurements were obtained by placing a 8 × 8 mm region of interest in the subendocardial basal anteroseptum, posterior wall, and pericardium at the peak of the R-wave on the ECG in the parasternal long-axis view. cIB was obtained by subtracting average pericardial backscatter intensity from average myocardial backscatter intensity of the anteroseptum or posterior wall.

### Statistical analysis

A linear mixed model was used to assess the differences in change in LA and LV dimensions and function over the follow-up period between the three groups as it accounts for the correlation of repeated measurements over time within patients. The group (1, 2, or 3) and timing of echocardiogram (baseline or 1 year) were incorporated in the model as fixed variables in addition to the interaction between the group and timing of echocardiogram. Restricted maximum likelihood estimation with an unstructured covariance matrix and a random intercept model was used. The estimated marginal means and 95 % confidence interval were presented. Non-Gaussian continuous variables, such as LDL, triglycerides, CRP, indexed LA volumes, mitral E-wave velocity, E/e’, and E/A, were transformed as appropriate. Pairwise comparisons were performed using Bonferroni correction. The Kruskal–Wallis test was used to compare MRC dyspnea grade between groups at baseline, and over time. A two-sided *p* value <0.05 was considered significant. Statistical analyses were performed using STATA v12 (STATA Corporation, Texas).

## Results

### Baseline clinical, metabolic, and echocardiographic characteristics

The baseline clinical and metabolic characteristics of the three groups of patients are summarized in Table 1. The three groups of patients were comparable in all their clinical and metabolic parameters except that Group 3 patients were heaviest and had the highest body mass index (BMI). Metabolic profiles were also comparable except Group 2 had the higher total cholesterol compared with Group 1. In particular, there were no significant differences in the baseline HbA1c between the three groups. Group 3 patients had the highest, though nonsignificant, baseline CRP levels. The median MRC dyspnea grade was 2 in all three groups at baseline (*p* = 0.798). Medication use at baseline is presented in Table 2.

**Table 1:** Baseline and follow-up clinical and metabolic characteristics in the three groups of patients

Characteristic	Group 1: no weight loss + worse glycaemic control (*n* = 20)	Group 2: no weight loss + improved glycaemic control (*n* = 20)	Group 3: weight loss + improved glycaemic control (*n* = 10)
Body mass index (kg/m^2^)			
Baseline	34.4 (31.2–37.5)	31.5 (28.3–34.7)	44.3 (39.8–48.7)^§†^
Follow-up	35.7 (32.5–38.8)	32.5 (29.4–35.7)*	34.5 (30.0–40.0)*
Weight (kg)			
Baseline	92.1 (82.1–102.0)	81.3 (71.3–91.2)	123.6 (109.5–137.7)^§†^
Follow-up	94.2 (85.1–103.3)*	84.3 (75.2–93.4)*	95.7 (82.8–108.5)*
Waist circumference (cm)			
Baseline	109 (102, 115)	103 (96, 109)^§^	128 (119, 137)^†^
Follow-up	111 (104, 117)	105 (99, 111)	114 (106, 122)*
Waist-to-hip ratio			
Baseline	0.94 (0.91–0.98)	0.95 (0.91–0.99)	0.94 (0.89–0.99)
Follow-up	0.94 (0.91–0.96)	0.96 (0.93–0.99)	0.96 (0.92–1.00)
Systolic blood pressure (mmHg)			
Baseline	137 (129–144)	133 (125–140)	128 (117–138)
Follow-up	134 (128–140)	129 (123–135)	131.4 (123–140)
Diastolic blood pressure (mmHg)			
Baseline	78 (73–82)	73 (68–78)	74 (68–81)
Follow-up	81 (77–86)	77 (72–81)	79 (72–85)
HbA1c, NGSP (%) IFCC (mmol/mol)			
Baseline	9.4 (8.6–10.2)	9.9 (9.1–10.7)	9.5 (8.4–10.6)
	79 (70–88)	85 (76–93)	80 (68–92)
Follow-up	9.5 (8.7–10.3)	7.3 (6.5–8.1)*^†^	6.7 (5.5–7.1)*^†^
	80 (72–89)	56 (48–65)	50 (37–54)
Total cholesterol (mg/dL)			
Baseline	5.2 (4.7–5.7)	4.1 (3.5–4.6)^†^	5.0 (4.3–5.8)
Follow-up	4.8 (4.3–5.4)	3.8 (3.2–4.3)^†^	4.6 (3.9–5.4)
LDL cholesterol (mg/dL)			
Baseline	2.7 (2.2–3.3)	1.8 (1.5–2.1)^†^	2.6 (2.0–3.4)
Follow-up	2.3 (1.8–2.1)	1.8 (1.4–2.2)	2.5 (1.8–3.3)
HDL cholesterol (mg/dL)			
Baseline	1.2 (1.1–1.4)	1.3 (1.2–1.4)	1.09 (0.9–1.3)
Follow-up	1.2 (1.1–1.3)	1.2 (1.1–1.3)	1.2 (1.1–1.4)*
Triglycerides (mg/dL)			
Baseline	1.7 (1.4–2.2)	1.3 (1.1–1.6)	2.2 (1.5–3.2)
Follow-up	1.7 (1.4–2.2)	1.2 (1.0–1.5)	1.4 (1.1–1.9)
eGFR (mL/min/1.73 m^2^)			
Baseline	91 (83–99)	92 (84–99)	89 (78–100)
Follow-up	101 (93–110)	84 (75–92)	96 (84–108)
C-reactive protein (mg/L)			
Baseline	3.6 (2.2–5.9)	3.0 (1.8–5.1)	10.2 (5.2–19.8)
Follow-up	2.8 (1.8–4.4)	2.7 (1.7–4.4)	4.4 (2.3–8.4)

Estimates from a linear mixed model. Data are presented as estimated marginal means and 95 % confidence interval

*NGSP* National glycohaemoglobin standardization programme

Within groups: * *p* < 0.05 for 1-year follow-up versus baseline

Between groups: ^§^ *p* < 0.001 versus Group 2; ^†^ *p* < 0.05 versus Group 1

**Table 2:** Baseline medications in the three groups of patients

Medication	Group 1: no weight loss + worse glycaemic control (*n* = 20)	Group 2: no weight loss + improved glycaemic control (*n* = 20)	Group 3: weight loss + improved glycaemic control (*n* = 10)
Aspirin or clopidogrel, *n* (%)	10 (25 %)	16 (40 %)	4 (20 %)
Angiotensin converting enzyme inhibitor or angiotensin-II receptor blocker, *n* (%)	32 (80 %)	26 (65 %)	16 (80 %)
Calcium channel blocker, *n* (%)	0 (0 %)	6 (15 %)	6 (30 %)
Beta-blocker, *n* (%)	2 (5 %)	4 (10 %)	0 (0 %)
Diuretic, *n* (%)	6 (15 %)	2 (5 %)	4 (20 %)
Spironolactone, *n* (%)	0 (0 %)	2 (5 %)	0 (0 %)
Statin, *n* (%)	28 (70 %)	26 (65 %)	10 (50 %)
Sulfonylurea, *n* (%)	6 (15 %)	20 (50 %)	6 (30 %)
Biguanide, *n* (%)	38 (95 %)	32 (80 %)	18 (90 %)
Di-peptidyl peptidase-4 inhibitor , *n* (%)	10 (26 %)	8 (20 %)	4 (20 %)
Insulin, *n* (%)	20 (50 %)	16 (40 %)	10 (50 %)

The baseline echocardiographic characteristics are listed in Table 3. The three groups had similar LV dimensions, wall thickness, ejection fraction, and diastolic function measured by the septal e’ velocities and diastolic function grades. The LV anterior septal wall thickness was highest in Group 3, but the LV mass indices were comparable. Patients in Group 3 had the most impaired LV systolic function as measured by LV GLS and strain rate. The cIB of the LV anteroseptal wall was highest in Group 3. All measures of LA function were similar across the three groups at baseline.

**Table 3:** Baseline and follow-up echocardiographic characteristics in the three groups of patients

Characteristic	Group 1: no weight loss + worse glycaemic control (*n* = 20)	Group 2: no weight loss + improved glycaemic control (*n* = 20)	Group 3: weight loss + improved glycaemic control (*n* = 10)
LV mass index (g/m^2^)			
Baseline	78.9 (68.6–89.3)	93.5 (83.1–103.9)	90.1 (75.4–104.8)
Follow-up	83.3 (75.5–91.0)	89.2 (81.4–97.0)	75.6 (64.6–86.5)
LV end diastolic volume (mL)			
Baseline	67 (58–80)	66 (54–78)	89 (72–106)
Follow-up	71 (60–82)	64 (53–76)	91 (75–107)
LV end systolic volume (mL)			
Baseline	24 (18–31)	27 (20–34)	36 (26–45)
Follow-up	23 (18–27)	20 (15–24)	29 (22–35)
LVEF (%)			
Baseline	65 (62–69)	61 (58–65)	60 (54–65)
Follow-up	69 (66–72)	70 (66–73)*	69 (64–73)*
E (cm/s)			
Baseline	74 (67–83)	67 (61–75)	70 (61–82)
Follow-up	80 (72–88)	75 (68–82)	73 (64–83)
A (cm/s)			
Baseline	87 (78–96)	72 (63–81)	82 (70–95)
Follow-up	88 (80–96)	73 (66–81)	80 (69–91)
E/A			
Baseline	1.06 (0.98–1.13)	1.03 (0.95–1.10)	1.07 (0.96–1.18)
Follow-up	1.06 (0.99–1.12)	1.00 (0.93–1.07)	1.07 (0.97–1.17)
Septal s’ (cm/s)			
Baseline	7.0 (6.4–7.6)	6.5 (5.9–8.1)	6.1 (5.3–6.9)
Follow-up	6.8 (6.3–7.3)	6.5 (6.0–7.0)	6.5 (5.7–7.3)
Septal e’ (cm/s)			
Baseline	6.3 (5.8–6.7)	6.1 (5.6–6.6)	5.9 (5.2–6.6)
Follow-up	6.6 (5.7–7.4)	7.5 (6.6–8.3)*	8.2 (7.0–9.4)*
Septal a’ (cm/s)			
Baseline	9.6 (8.5–10.6)	8.8 (7.7–9.8)	8.8 (7.3–10.3)
Follow-up	9.2 (8.3–10.1)	8.1 (7.2–9.0)	8.2 (7.0–9.4)
Septal E/e’			
Baseline	11.9 (10.7–13.5)	11.1 (10–12.5)	11.9 (10.2–14.2)
Follow-up	12.3 (10.7–14.5)	10.2 (9.1–11.6)	9.0 (7.8–10.7)*
LA dimension (mm)			
Baseline	35 (33, 38)	35 (33, 38)	39 (46, 42)
Follow-up	36 (34, 38)	33 (31, 35)	33 (30, 36)*
LA maximum volume indexed (mL/m^2^)			
Baseline	26 (24–28)	28 (26–32)	28 (24–32)
Follow-up	27 (24–31)	28 (25–31)	30 (25–36)
LA active emptying volume indexed (mL/m^2^)			
Baseline	10 (9–12)	10 (8–11)	9 (7–11)
Follow-up	10 (8–11)	10 (8–11)	12 (10–14)
LA passive emptying volume indexed (mL/m^2^)			
Baseline	9 (8–11)	11 (9–13)	13 (11–15)
Follow-up	11 (9–14)	11 (9–13)	11 (8–14)
Anteroseptal wall cIB (dB)			
Baseline	−14.1 (−16.6 to 11.6)	−10.6 (−13.1 to 8.1)	−6.0 (−9.6 to 2.5)†
Follow-up	−14.1 (−16.4 to 11.8)	−12.5 (−14.8 to 10.2)	−13.1 (−16.4 to 9.8)*
Posterior wall cIB (dB)			
Baseline	−16.5 (−19.0 to 14.1)	−12.1 (−14.5 to 9.6)	−11.7 (−15.2 to 8.3)
Follow-up	−16.6 (−18.8 to 14.5)	−15.3 (−17.4 to 13.2)	−13.9 (−16.9 to 10.9)*
LV global longitudinal systolic strain (%)			
Baseline	−17.3 (−18.6 to 16.0)	−15.3 (−16.6 to 14.0)	−13.0 (−14.8 to 11.2)†
Follow-up	−17.9 (−19.0 to 16.8)	−19.4 (−20.5 to 18.3)*	−19.3 (−18.9 to 17.8)*
LV global longitudinal systolic strain rate (1/s)			
Baseline	−0.99 (−1.06 to 0.91)	−0.82 (−0.89 to 0.74)†	−0.77 (−0.87 to 0.66)†
Follow-up	−0.98 (−1.05 to 0.91)	−1.03 (−1.1 to 0.96)*	−0.99 (−1.1 to 0.89)*

Estimates from a linear mixed model. Data are presented as estimated marginal means and 95 % confidence interval

Within groups: * *p* < 0.05 for 1-year follow-up versus baseline

Between groups: ^†^ *p* < 0.05 versus Group 1

### Follow-up clinical and echocardiographic characteristics

Table 1 shows the follow-up clinical and metabolic characteristics of the three groups of patients. By study design, the body weights, BMI, and HbA1c of patients in Group 3 decreased significantly but that of Group 1 remained the same at 12 months. There was also an increase in HDL-C, and a marginal decrease in triglycerides in Group 3. There was a significant improvement in MRC dyspnea grade from baseline to follow-up in Group 2 (grade 2 vs. grade 1, *p* = 0.0003) and Group 3 (grade 2 vs. grade 1, *p* = 0.001), but not Group 1 (grade 2 vs. grade 2, *p* = 0.483).

**Fig. 1:**
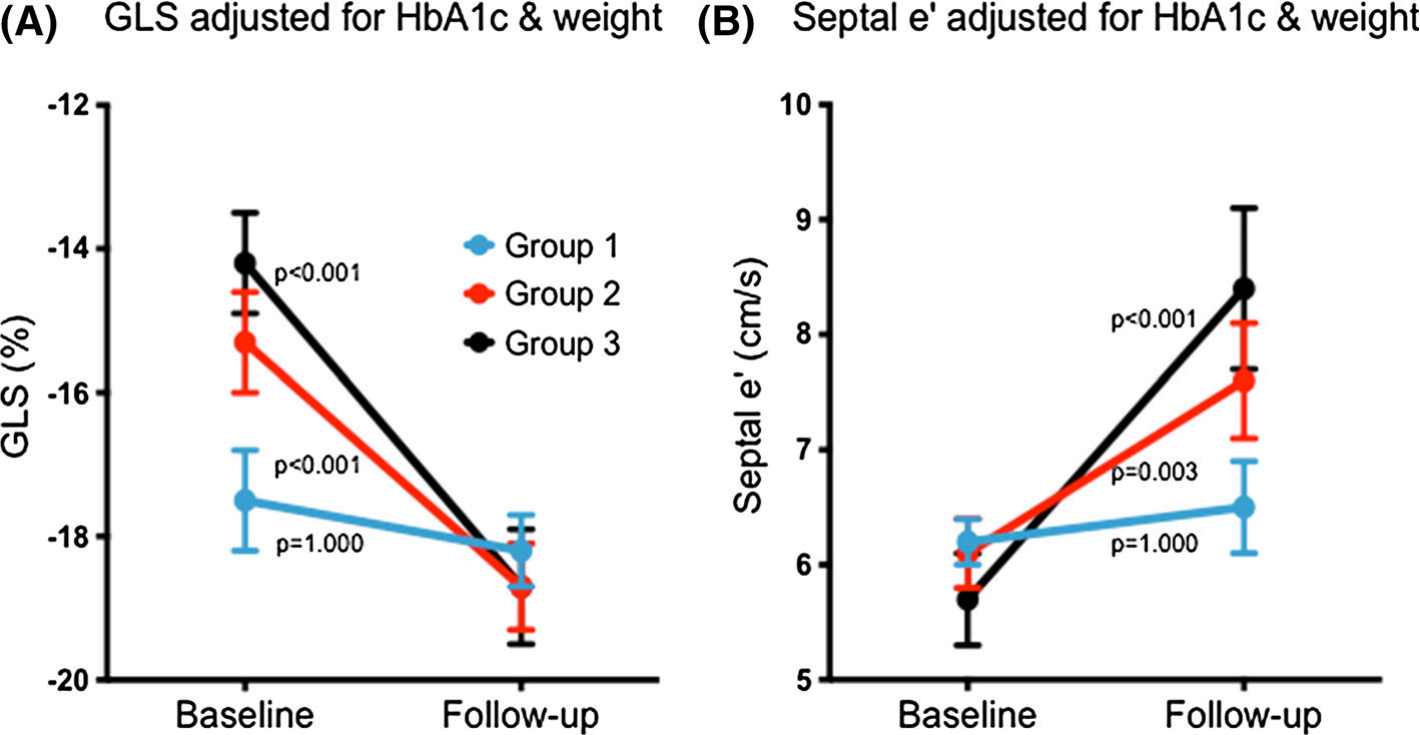
Changes in left ventricular global longitudinal strain and septal e’ by group. An improvement in GLS and septal e’ can be seen in Groups 2 and 3. No improvement in these parameters is noted in Group 1. Estimates from a linear mixed model. Data are presented as estimated marginal mean and 95 % confidence interval. Analysis adjusted for the corresponding baseline variable, changes in HbA1c and weight

Table 3 shows the follow-up echocardiographic characteristics. By 12 months, there was a reduction in LV anteroseptal wall thickness in Group 3 and a reduction in LV end systolic volume leading to an increase in LV ejection fraction in Groups 2 and 3. There was an increase in septal e’ velocities (Fig. 1) and a reduction in septal E/e’ ratio in Groups 2 and 3. The E/e’ ratio in Group 1 actually increased. There was a reduction in the LV anteroseptal wall cIB in Group 3 only. There was a significant improvement in LV GLS and strain rate in Groups 2 and 3 with the patients in Group 3 experiencing the larger improvement (Fig. 1). The LV GLS was worst in Group 3 at baseline but was comparable between Groups 2 and 3 at follow-up.

### Inter- and intra-observer variability

Left ventricular GLS measurements were repeated in ten randomly selected patients by the same observer (ML) on the same echocardiographic images, and by a second observer (DL) to determine intra-observer and inter-observer variability, respectively. The intra-observer mean differences for LV GLS were −0.19 ± 0.36 % (*r* = 0.994). The inter-observer mean differences were −0.33 ± 1.02 % (*r* = 0.94).

## Discussion

The present study evaluated three groups of age-, gender-, and baseline HbA1c-matched patients with type 2 diabetes who had subclinical LV systolic and diastolic dysfunction. They received intervention by diet and exercise advice, anti-hyperglycaemic medications, and/or surgery. The resultant improvement in glycaemic control led to significant improvements in both systolic function (LV GLS and ejection fraction) and diastolic function (*e*’). Furthermore, weight loss in addition to improved glycaemic control (Group 3) resulted in the largest improvements in LV systolic and diastolic function, despite having worst function at baseline. Furthermore, there was a reduction in cIB in this group reflecting a decrease in myocardial interstitial fibrosis. Our study demonstrates the beneficial and additive effects of improved glycaemic control and weight loss in improving cardiac function in overweight patients with type 2 diabetes.

### Left ventricular systolic and diastolic function in diabetes and impact of therapeutic intervention

The presence of LV dysfunction has been well described in patients with type 2 diabetes independent of myocardial ischaemia or hypertension [1–3]. We evaluated LV GLS and e’ velocities in our patients as measures of LV systolic and diastolic function, respectively. These measures have proven advantages over LV ejection fraction and mitral E and A velocities as measures of LV systolic and diastolic function, respectively. In addition, both LV GLS and e’ velocities have incremental prognostic value over a wide range of cardiovascular diseases [22, 28].

The underlying pathophysiologic mechanisms of diabetic cardiomyopathy are multifactorial and hyperglycaemia plays a central role [6] Furthermore, patients with type 2 diabetes are often obese and obesity per se has been linked to LV dysfunction [29].

There is increasing evidence to suggest a link between glycaemic control and LV function in diabetes. Studies have suggested poor glycaemic control was associated with LV diastolic dysfunction, manifest as either lower e’ velocities or raised LV filling pressure; and/or more impaired LV systolic function with lower systolic strain [2, 8, 9]. Furthermore, we have recently demonstrated that improving glycaemic control in such patients resulted in improvement in LV systolic and diastolic function [30]. The largest improvements in the aforementioned study were seen in patients with the largest reduction in HbA1c levels, and in those with the lowest HbA1c levels at the end of the study. In contrast, patients who had worsened glycaemic control experienced further deterioration of LV systolic function. However, studies examining the additive effects of weight loss and improved glycaemic control on LV function changes have been lacking.

Weight loss, however achieved, has been shown to result in improved LV systolic and diastolic function [31]. In a study of 261 patients with BMI ≥ 30 kg/m^2^ who undertook a behavioural intervention programme including dietary restriction and exercise training, independent predictors of improvement in LV function were weight reduction, improvement in insulin resistance and absence of diabetes [32]. There was no significant reduction in HbA1c levels with intervention in either the adherent group or in the non-adherent group; therefore, the effects of improved glycaemic control on LV function was not examined. Caloric restriction in obese patients with type 2 diabetes led to improved LV diastolic function [33]. The severely obese patients have greater inflammatory burden suggested by the significantly higher C-reactive protein levels seen in patients in Group 3. While bariatric surgery has proven benefits in weight reduction in obese patients which leads to improvement in LV systolic and diastolic function [34, 35], it is a different treatment approach to dieting and exercise with additional impact on the neuro-hormonal axis. Patients who have undergone sleeve gastrectomy have been found to have reduced ghrelin levels, while the hindgut theory proposes that rapid delivery of undigested nutrients to the hindgut following bariatric surgery up-regulates production of glucagon-like peptide 1 and peptide-YY [36, 37]. How these neuro-hormonal changes contribute to improvement in diabetic cardiomyopathy is unclear. In a study of severely obese patients, beneficial effects of weight loss with bariatric surgery on ECG abnormalities were seen more often in those who lost weight and achieved normotension [38]. This supports the importance of targeting multiple co-morbidities in these patients to achieve desirable therapeutic goals.

Our study underlines the importance of both weight loss and improving glycaemic control for the improvement of cardiac function in obese patients with type 2 diabetes. In looking after subjects with type 2 diabetes, clinicians are often faced with the task of managing their obesity as an adjunct to improve their glycaemic control. Many therapies for diabetes such as insulin and thiazolidinediones may improve glycaemic control, but these agents can increase the weight of patients. Poor glycaemic control and obesity are both associated with worsening of LV function, and targeting both parameters may have important and independent effects in preventing the development of diabetic cardiomyopathy. Given the current obesity epidemic and prevalence of obesity and diabetic cardiomyopathy, these results have important implications for preventing obesity and diabetes-related morbidity and mortality.

### Limitations

Our study was not a randomized trial. Such a study would have been unethical. Given that the goal of our study was to better understand the contributions of weight loss and glycaemic improvement towards modulating LV function, we used a group of patients with no weight loss and no improvement in glycaemic control as a reference category, and these patients may have been inherently different from subjects in the other two groups. We did not have a group of patients who achieved weight loss without any reduction in HbA1c levels to examine the independent contribution of weight loss alone without improved glycaemic control on LV function. However, as the study by Kosmala et al. has shown [32], improvement in LV function with weight loss alone (their patients did not have significant reduction in HbA1c with behavioural intervention) was less frequently observed in patients with diabetes. We did not evaluate changes in insulin resistance in our patients. Weight loss in our Group 3 patients was achieved with sleeve gastrectomy. The addition of a further group of patients who were able to both improve glycaemic control and lose weight by non-surgical means, and who are matched to group 1 and 2 for age, gender, BMI, and baseline HbA1c would be ideal. Unfortunately, we do not have such a group of patients in this study. This, together with the high non-adherence rate in the Kosmala study, highlight the significant challenges in achieving significant weight loss in patients with type 2 diabetes and obesity, despite an intensive and supervised multidisciplinary approach. Finally, for patients who are only overweight or only mildly obese, we cannot be certain whether weight loss by diet and exercise alone and better glycaemic control confer additive benefits.

## Conclusions

In overweight patients with type 2 diabetes, weight loss and improved glycaemic control had additive beneficial effects on improving LV systolic and diastolic function.
